# Artepillin C (3,5-diprenyl-4-hydroxycinnamic acid) sensitizes LNCaP prostate cancer cells to TRAIL-induced apoptosis

**DOI:** 10.3892/ijo.2012.1527

**Published:** 2012-06-25

**Authors:** EWELINA SZLISZKA, GRZEGORZ ZYDOWICZ, ELZBIETA MIZGALA, WOJCIECH KROL

**Affiliations:** 1 Departments of Microbiology and Immunology and; 2 Family Medicine, Medical University of Silesia in Katowice, Zabrze, Poland

**Keywords:** artepillin C, green propolis, tumour necrosis factor-related apoptosis-inducing ligand, apoptosis, prostate cancer, chemoprevention

## Abstract

Naturally occurring phenolic compounds have been shown to sensitize prostate cancer cells to tumour necrosis factor-related apoptosis-inducing ligand (TRAIL)-induced apoptosis. TRAIL is a potent stimulator of apoptosis in cancer cells and an important immune effector molecule in the surveillance and elimination of developing tumours. However, many cancer cells are resistant to TRAIL-mediated death. In this study, we aimed to determine the mechanisms by which TRAIL resistance can be overcome in prostate cancer cells by 3,5-diprenyl-4-hydroxycinnamic acid (artepillin C). Artepillin C is a bioactive component of Brazilian green propolis that possesses antitumour and chemopreventive activities. TRAIL-resistant LNCaP prostate cancer cells were treated with TRAIL and artepillin C. Cytotoxicity was measured by MTT and lactate dehydrogenase (LDH) assays. Apoptosis was detected using Annexin V-FITC staining by flow cytometry and fluorescence microscopy. Death receptor (DR) (TRAIL-R1/DR4 and TRAIL-R2/DR5) expression was analyzed using flow cytometry. Mitochondrial membrane potential (ΔΨm) was evaluated using DePsipher staining by fluorescence micro scopy. The inhibition of NF-κB (p65) activation was confirmed with the ELISA-based TransAM NF-κB kit. Caspase-8 and caspase-3 activities were determined by colorimetric protease assays. The results showed that artepillin C sensitized the TRAIL-resistant LNCaP cells by engaging the extrinsic (receptor-mediated) and intrinsic (mitochondrial) apoptotic pathways. Artepillin C increased the expression of TRAIL-R2 and decreased the activity of NF-κB. Co-treatment with TRAIL and artepillin C induced the significant activation of caspase-8 and caspase-3, as well as the disruption of ΔΨm. These findings show that prostate cancer cells can be sensitized to TRAIL-mediated immunoprevention by artepillin C and confirm the role of phenolic compounds in prostate cancer immunochemoprevention.

## Introduction

The induction of cancer cell-specific apoptosis via the activation of tumour necrosis factor-related apoptosis-inducing ligand (TRAIL) signalling has become an important focus of cancer research. TRAIL triggers apoptosis in cancer cells with no toxicity toward normal tissues (1,2). Endogenous TRAIL plays an important role in immunosurveillance and defence against tumour cells. TRAIL-mediated apoptosis of pre-malignant or malignant cells represents an immune preventive mechanism against tumour initiation, formation and progression. TRAIL is expressed on the surface of T lymphocytes, natural killer cells, dendritic cells, neutrophils, monocytes or macrophages and can be cleaved into a soluble, secreted form (1–3). This death ligand induces apoptosis in cancer cells via a receptor-mediated pathway involving interactions with TRAIL-R1/death receptor (DR)4 and/or TRAIL-R2/DR5. Stimulation of the death receptor system by TRAIL results in the recruitment of the adaptor molecule, Fas-associated death domain (FADD), to form the death inducing signalling complex (DISC), which subsequently activates caspase-8. Crosstalk between the extrinsic (receptor-dependent) and intrinsic (mitochondrial-dependent) apoptotic pathways is linked by caspase-8. The activation of caspase-8 directly causes the activation of the caspase cascade and cell death. Simultaneously, caspase-8 leads indirectly to the activation of effector caspases through the cleavage of the BH3-interacting domain death agonist (Bid), along with the release of cytochrome *c* and mitochondrial membrane potential (ΔΨm) disruption (1–6). However, some cancer cells are resistant to TRAIL-induced cytotoxicity (7–11). This failure to undergo apoptosis has been implicated in the resistance of cancer cells to TRAIL surveillance and, therefore, in tumour development (1–3,6). The expression of the death receptors and pro-apoptotic or anti-apoptotic proteins in cancer cells is involved in TRAIL resistance (1,2,6). TRAIL-resistant prostate cancer cells can be sensitized to TRAIL-mediated apoptosis by certain phenolic compounds (6,8,12–20).

**Figure 1:**
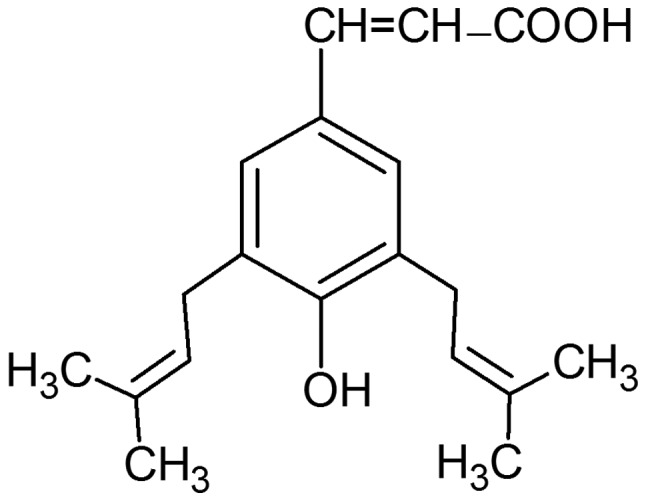
Chemical structure of artepillin C (3,5-diprenyl-4-hydroxycinnamic acid).

Artepillin C (3,5-diprenyl-4-hydroxycinnamic acid) is the major biologically active phenolic component found in green propolis, which is collected from the plant *Baccharis dracunculiforia* in Southeastern Brazil (21–23). Artepillin C possesses antioxidant, antimicrobial, anti-inflammatory, antigenotoxic, anti-angiogenic and anticancer properties (24–36). The structure of this hydroxycinnamic acid derivative is presented in Fig. 1. Artepillin C exerts direct antiproliferative, cytotoxic and apoptotic effects on gastric, colon or lung cancer cells and inhibits the growth of transplanted solid human or mouse tumours in athymic and thymic mice, respectively (25). The role of natural phenolic compounds in cancer prevention has been confirmed in numerous laboratory and epidemiological studies (37–43).

Prostate cancer is one of the most commonly diagnosed cancers in men, the third leading cause of cancer-related mortality in Europe, and the second in the United States (16,44). Chemopreventive intervention using dietary phenolics is an attractive option in prostate cancer due to its incidence, prevalence and disease-related morbidity and mortality (45).

Previous findings have demonstrated that the ethanolic extract of Brazilian green propolis (EEP) and its constituent, artepillin C, can help cells overcome TRAIL resistance and significantly augments the apoptotic activity of TRAIL in LNCaP prostate cancer cells (17). Artepillin C sensitizes prostate cancer cells to TRAIL-mediated immunoprevention, which confirms the potential role of this prenylated hydroxycinnamic acid derivative as a chemopreventive agent in prostate carcinogenesis. The aim of this study was to evaluate the mechanisms by which artepillin C affects the TRAIL-induced death signalling pathway in prostate cancer cells.

## Materials and methods

### Prostate cancer cell culture

The LNCaP human androgen-dependent prostate cancer cell line was obtained from the German Collection of Microorganisms and Cell Cultures (DSMZ, Braunschweig, Germany). The cells were grown in monolayer cultures at 37°C in a 5% CO_2_ humidified incubator and were maintained in RPMI-1640 medium supplemented with 10% heat-inactivated fetal bovine serum (FBS), 4 mM L-glutamine, 100 U/ml penicillin and 100 *μ*g/ml streptomycin (7,8,16,17). Reagents for cell culture were purchased from the PAA Cell Culture Company (Pasching, Austria).

### Reagents

Artepillin C was provided by Wako Pure Chemicals (Osaka, Japan) as a natural component isolated from Brazilian green propolis. Artepillin C was dissolved in dimethyl sulphoxide (DMSO) to obtain the working concentrations. Soluble recombinant human TRAIL was purchased from PeproTech Inc. (Rocky Hill, NJ, USA). Human recombinant TRAIL-R1/Fc and TRAIL-R2/Fc chimera proteins, the general caspase inhibitor, Z-VAD-FMK, the caspase-8 inhibitor, Z-IETD-FMK, and the caspase-3 inhibitor, Z-DEVD-FMK, were obtained from R&D Systems (Minneapolis, MN, USA).

### Cytotoxicity assay

Cytotoxicity was measured by the 3-(4,5- dimethyl-2-thiazyl)-2,5-diphenyl-2H-tetrazolium bromide (MTT) assay (46,47). The MTT assay is based on the cleavage of the tetrazolium salt MTT to a blue formazan dye by viable cells. LNCaP cells (2×10^5^/ml) were seeded in a 96-well plate for 24 h before the experiments. The cells were then incubated with TRAIL (50–200 ng/ml) and/or artepillin C (50–100 *μ*M). After 24 h, 20 *μ*l of MTT solution (5 mg/ml) were added to each well for 4 h. The resulting blue formazan crystals were dissolved in DMSO. These reagents were purchased from Sigma Chemical Company (St. Louis, MO, USA). The controls included native cells and medium alone. Spectrophotometric absorbance was measured at a 550-nm wavelength using a microplate reader (ELx 800, Bio-Tek Instruments Inc., Winooski, VT, USA). The percentage cytotoxicity was calculated by the following formula: percentage cytotoxicity (cell death) = [1−(absorbance value of experimental wells/absorbance value of control wells)]) ×100%.

### Lactate dehydrogenase (LDH) release assay

LDH is a stable cytosolic enzyme that is released upon membrane damage in necrotic cells. LDH activity was measured using a cytotoxicity assay kit (Roche Diagnostics GmbH, Mannheim, Germany) (14,48). LNCaP cells were treated with TRAIL (50–200 ng/ml) and/or artepillin C (50–100 *μ*M) for the indicated period of time. LDH released into the culture supernatants was detected with a coupled enzymatic assay, that results in the conversion of a tetrazolium salt into a red formazan product. Spectrophotometric absorbance was measured at a 490-nm wavelength using a microplate reader. Maximal release of LDH was obtained after treating the control cells with 1% Triton X-100 (Sigma) for 10 min at room tempe rature. The percentage of necrotic cells was expressed using the following formula: (sample value/maximal release) ×100%.

### Detection of apoptosis by flow cytometry

Apoptosis was determined by flow cytometry using the Apoptest-FITC kit with Annexin V (Dako, Glostrup, Denmark). LNCaP cells (2×10^5^/ml) were seeded in 24-well plates for 24 h prior to experimentation and then exposed to TRAIL (100 ng/ml) and/or artepillin C (50–100 *μ*M) for 24 h. After this incubation, the cells were washed twice with phosphate-buffered saline (PBS) solution and resuspended in 1 ml of binding buffer. The cell suspension (500 *μ*l) was then incubated with 5 *μ*l of Annexin V-FITC and 10 *μ*l of propidium iodide (PI) for 10 min at room temperature in the dark. The population of Annexin V-positive cells was evaluated by flow cytometry (BD FACScan; Becton-Dickinson Immunocytometry Systems, San Jose, CA, USA) (15,49).

### Detection of apoptosis by fluorescence microscopy

Apoptotic cells were quantified using the fluorescence microscopy method of the Apoptotic & Necrotic & Healthy Cells Quantification kit from Biotium, Inc. (Hayward, CA, USA) (15,17). LNCaP cells (2×10^5^/ml) were seeded in a 24-well plate for 24 h before the experiments. TRAIL (100 ng/ml) and/or artepillin C (50 and 100 *μ*M) were added to the cells, and 24 h later, the cells were washed with PBS and trypsinised. The cells were then centrifuged to discard the supernatant, washed with PBS and resuspended in binding buffer (100 *μ*l/sample). A combination of 5 *μ*l of Annexin V-FITC, 5 *μ*l of ethidium homodimer III and 5 *μ*l of Hoechst 33342 solution was added to each tube. The samples were incubated at room temperature for 15 min in the dark, and then the cells were washed with binding buffer, placed on a glass slide and covered with a glass cover slip. The stained cells were observed with an IX51 fluorescence inverted microscope (Olympus, Tokyo, Japan) using filter sets for FITC, TRITC and DAPI. The cells were counted, and the number of apoptotic cells is expressed as a percentage of the total number of cells.

### Flow cytometric analysis of death receptor expression on the cell surface

The cell surface expression of the death receptors, TRAIL-R1 and TRAIL-R2, was determined by flow cytometry (BD FACSCanto, Becton-Dickinson Immunocytometry Systems). LNCaP cells (2×10^5^/ml) were seeded in 24-well plates for 24 h and exposed to artepillin C (50 and 100 *μ*M) for 24 h. The cells were then harvested using a solution of trypsin and ethylenediaminetetra acetic acid (EDTA), washed twice in PBS and resuspended in PBS containing 0.5% bovine serum albumin (BSA). LNCaP cells were incubated with 10 *μ*l phycoerythrin-conjugated anti-TRAIL-R1 or anti-TRAIL-R2 monoclonal antibody (R&D Systems) at 4°C for 45 min. After staining, the cells were washed with PBS and analyzed using flow cytometry (17–20,50). The control sample consisted of cells in a separate tube treated with phycoerythrin-labelled mouse IgG_1_ or mouse IgG_2B_ (R&D Systems).

### Caspase activity assays

Caspase-3 and caspase-8 activities were assessed using colorimetric protease assay kits (R&D Systems). The tests are based on the spectrophotometric detection of the chromophore, *p*-nitroaniline (pNA), after cleavage from the caspase substrate (caspase-specific peptide conjugated to pNA). LNCaP cells (1×10^6^/ml) were seeded in Petri dishes 24 h before the experiments. TRAIL (100 ng/ml) and/or artepillin C (50–100 *μ*M) were added to the cells, after 24 h the cells were centrifuged, the supernatant was discarded and the cells were treated with lysis buffer. The cell lysates were tested for protease activity by the addition of a labelled caspase substrate, DEVD-pNA for caspase-3 activity or IETD-pNA for caspase-8 activity. pNA absorbance was quantified using a V-630 spectrophotometer (Jasko International Co., Tokyo, Japan) at a 405-nm wavelength (20).

### Evaluation of ΔΨm by DePsipher staining

The DePsipher kit (R&D Systems) was used to measure the ΔΨm using fluorescence microscopy (17–20). LNCaP cells (2×10^5^/ml) were seeded in a 24-well plate 24 h prior to the experiments. TRAIL (100 ng/ml) and/or artepillin C (50–100 *μ*M) were added, and 24 h later, the cells were washed with PBS and harvested by trypsinisation. The cells were incubated in the dark with DePsipher (5,5′,6,6′-tetrachloro-1,1′,3,3′-tetraethylbenzimidazolyl carbocyanin iodide) solution at a concentration of 5 *μ*g/ml for 30 min at 37°C, washed with reaction buffer with stabiliser, placed on a glass slide and covered with a glass cover slip. The stained cells were observed with a fluorescence inverted microscope using filter sets for FITC and TRITC. DePsipher staining exhibits potential-dependent accumulation in the mitochondria, which is indicated by a fluorescence emission shift from red (590 nm) to green (530 nm).

### The activity of NF-κB

NF-κB activity was measured using the ELISA-based TransAM NF-κB kit (Active Motif Europe, Rixensart, Belgium) on nuclear extracts. LNCaP cells (1×10^6^/ml) were seeded in Petri dishes and allowed to attach for 24 h before the experiments. Artepillin C (50–100 *μ*M) with or without TRAIL (100 ng/ml) was added to the cells for 24 h. The commercially available Nuclear Extract kit was obtained from Active Motif Europe for the preparation of the LNCaP cell nuclear extracts. The TransAM NF-assay for NF-κB (p65) activity was performed according to the manufacturer’s instructions (17,19,20). NF-κB DNA-binding activity was assessed using the ELISA kit for the transcription factor, p65. Oligonucleotides containing the NF-κB consensus binding site (5′-GGGACTTCC-3′) were immobilised on a 96-well plate. The active forms of NF-κB in the nuclear extracts were bound to the oligonucleotides on the plate and detected colorimetrically. The samples were read at an absorbance of 450 nm on a spectrophotometer with a reference wavelength of 650 nm. The detection limit for the TransAM NF-κB kit is <0.4 ng/ml purified p65.

### Statistical analysis

The results are expressed as the means ± SD obtained from three independent experiments performed in quadruplicate (n=12) or duplicate (n=6). Statistical significance was evaluated using the Levene test or Bartlett χ^2^ test followed by analysis of variance (ANOVA) and post-hoc test. A p-value <0.05 was considered significant.

## Results

### Cytotoxic and apoptotic effects of TRAIL on LNCaP cells

**Figure 2:**
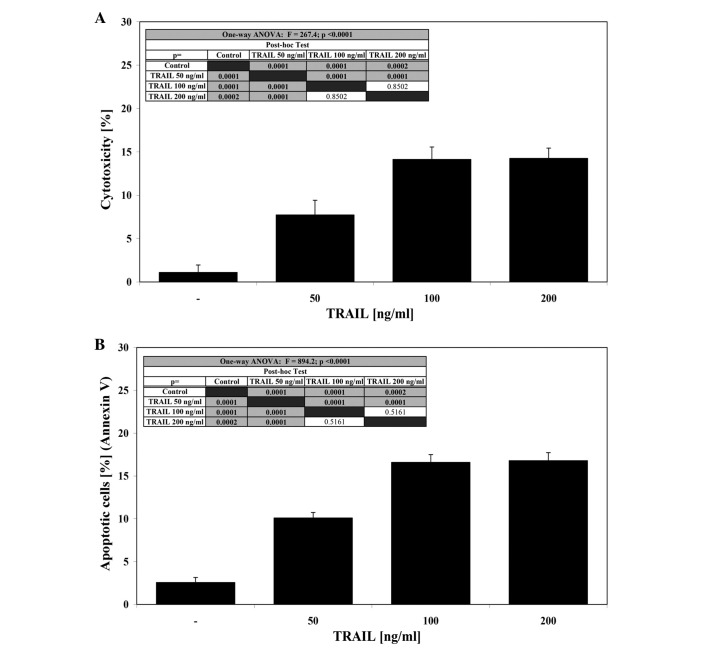
Cytotoxic and apoptotic effects of TRAIL on LNCaP prostate cancer cells. Cells were incubated for 24 h with 50–200 ng/ml TRAIL. (A) Cytotoxic activity of TRAIL in LNCaP cells. The percentage of cell death was measured using the MTT cytotoxicity assay. The values represent the means ± SD of three independent experiments performed in quadruplicate, n=12 (p<0.0001 as shown by ANOVA). (B) TRAIL-induced apoptosis in LNCaP cells. Apoptotic cell death was detected by flow cytometry using Annexin V-FITC staining. The values represent the means ± SD of three independent experiments performed in duplicate, n=6 (p<0.0001 as shown by ANOVA).

The cytotoxic effect of TRAIL at concentrations of 50–100 ng/ml after a 24-h incubation was 7.7±1.7–14.1±1.4% cell death (Fig. 2A). At the same concentrations TRAIL induced 10.1±0.6–16.6±0.9% apoptosis in a dose-dependent manner in LNCaP cells (Fig. 2B). TRAIL concentrations higher than 100 ng/ml resulted in no significant increase in cytotoxic or apoptotic activity. These data confirm that the LNCaP cell line is resistant to TRAIL-mediated apoptosis.

### Cytotoxic and apoptotic effects of TRAIL in combination with artepillin C on LNCaP cells

**Figure 3:**
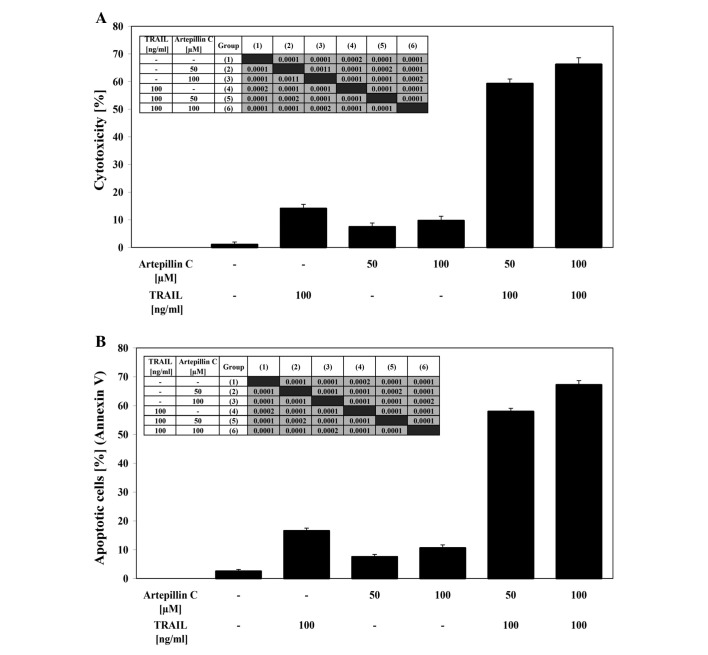

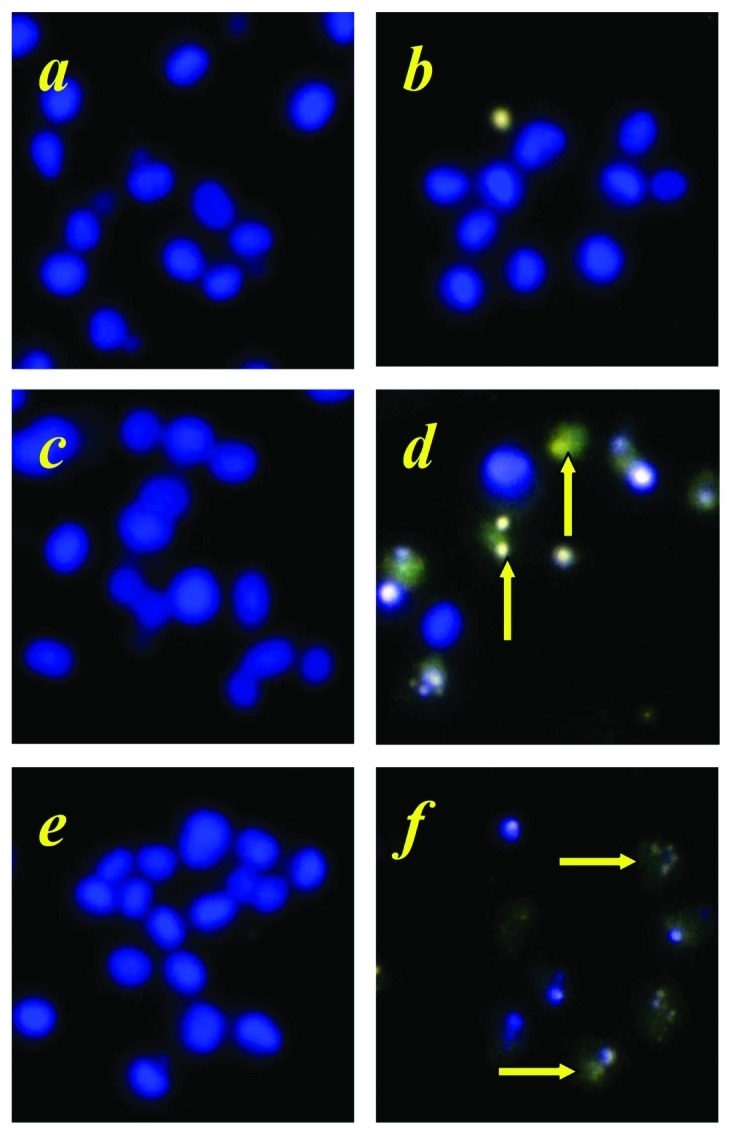
Cytotoxic and apoptotic effects of TRAIL in combination with artepillin C in LNCaP prostate cancer cells. Cells were incubated for 24 h with 100 ng/ml TRAIL and/or 50–100 *μ*M artepillin C. (A) Cytotoxic activity of TRAIL in combination with artepillin C in LNCaP cells. The percentage of cell death was measured using the MTT cytotoxicity assay. The values represent the means ± SD of three independent experiments performed in quadruplicate, n=12 (p<0.0001 TRAIL + artepillin C compared to TRAIL or artepillin C alone as shown by ANOVA). (B) TRAIL-induced apoptosis in combination with artepillin C in LNCaP cells. Apoptotic cell death was detected by flow cytometry using annexin V-FITC staining. The values represent the means ± SD of three independent experiments performed in duplicate, n=6 (p<0.0001 TRAIL + artepillin C compared to TRAIL or artepillin C alone as shown by ANOVA). (C) TRAIL-induced apoptosis in combination with artepillin C in LNCaP cells: (a) control cells, (b) cells incubated with 100 ng/ml TRAIL, (c) cells incubated with 50 *μ*M artepillin C, (d) cells incubated with 100 ng/ml TRAIL and 50 *μ*M artepillin C, (e) cells incubated with 100 *μ*M arte pillin C and (f) cells incubated with 100 ng/ml TRAIL and 100 *μ*M artepillin C. Apoptotic cell death was detected and visualised by fluorescence microscopy using Annexin V-FITC staining. Healthy cells (stained with Hoechst 33342) emitted blue fluorescence and apoptotic cells (stained with Hoechst 33342 and annexin V-FITC) emitted green and blue fluorescence (indicated by arrows).

After co-treatment of LNCaP cells with 100 ng/ml TRAIL and 50–100 *μ*M artepillin C for 24 h the cytotoxicity ranged from 59.3±1.6 to 66.3±2.3%. The cytotoxicity measured by MTT assay is shown in Fig. 3A. Artepillin C cooperated with TRAIL to induce apoptosis in the prostate cancer cells. When the cells were treated with the same concentration of TRAIL and artepillin C for 24 h, the percentage of apoptotic cells was elevated to 58.0±1.1–67.2±1.5% as determined by Annexin V-FITC staining using flow cytometry (Fig. 3B). Artepillin C sensitized the TRAIL-resistant LNCaP cells to TRAIL-mediated apoptosis. The necrotic cell death percentage of LNCaP cells examined by Apoptest-FITC and LDH assay was near zero. The Annexin V-FITC staining, visualised by fluorescence microscopy, supports the hypo thesis that the apoptotic activity of TRAIL was augmented by artepillin C in LNCaP cells (Fig. 3C).

### Effects of artepillin C on death receptor expression in LNCaP cells

**Figure 4:**
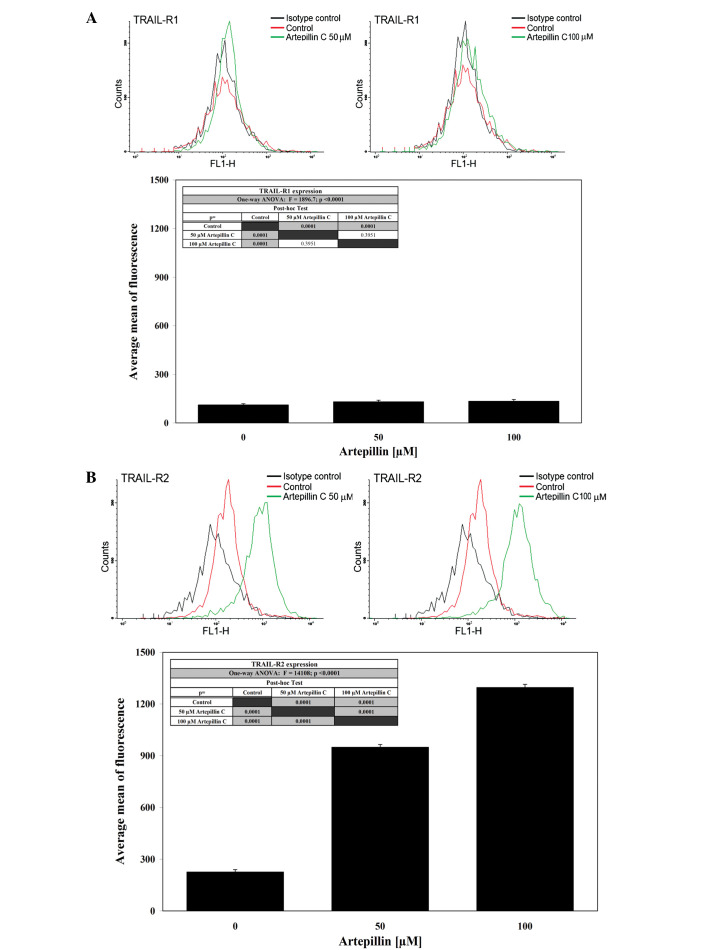
Effects of artepillin C on death receptor expression in LNCaP pro state cancer cells. Cells were incubated for 24 h with 50–100 *μ*M artepillin C. The surface expression of TRAIL-R1 and TRAIL-R2 on LNCaP cells was measured by flow cytometry. Representative histograms and the average mean fluorescence for (A) TRAIL-R1 expression and for (B) TRAIL-R2 expression are shown from three independent experiments performed in duplicate, n=6. The values represent the means ± SD (p<0.0001 artepillin C compared to control as shown by ANOVA).

The activation of death receptors on the cell surface is critical for TRAIL-mediated apoptosis. Therefore, we analyzed the expression of TRAIL-R1 and TRAIL-R2 in LNCaP cells after a 24-h treatment with 50–100 *μ*M artepillin C by flow cytometry (Fig. 4). Treatment with artepillin C significantly increased the expression of TRAIL-R2, but did not alter TRAIL-R1 expression on the cell surface. Artepillin C sensitized the prostate cancer cells through the extrinsic apoptotic pathway. To show that the induction of apoptosis caused by the co-treatment of TRAIL and artepillin C was mediated through TRAIL-R2, we used the TRAIL-R2/Fc chimera protein, which acts as a dominant-negative against endogenous TRAIL-R2. The chimeric protein efficiently blocked apoptosis when the cells were treated with TRAIL and artepillin C.

### Effects of TRAIL and artepillin C on caspase-8 and caspase-3 activities in LNCaP cells

**Figure 5:**
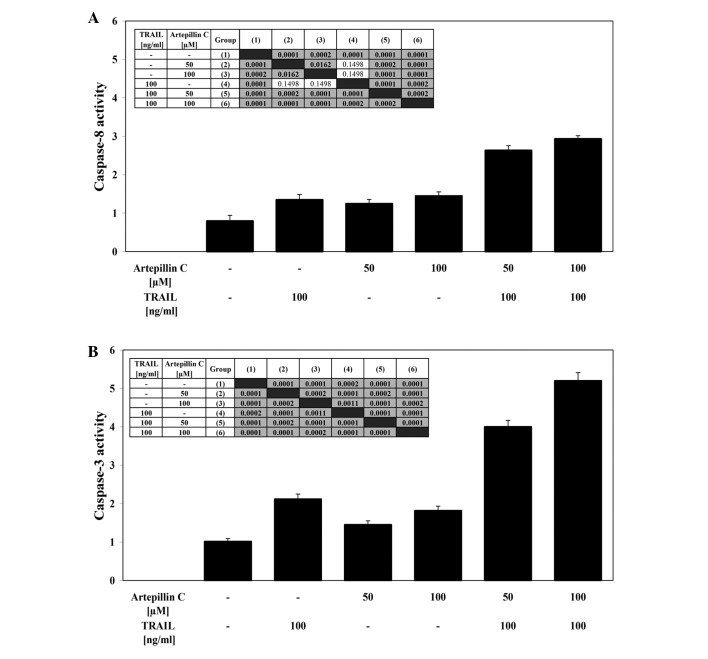
Effects of TRAIL in combination with artepillin C on caspase activities in LNCaP prostate cancer cells. Assessment of intracellular (A) caspase-8 and (B) caspase-3 activity in cells treated with 100 ng/ml TRAIL and/or 50–100 *μ*M artepillin C for 24 h. Caspase activities were measured using colorimetric protease assays based on the spectrophotometric detection of the chromophore, *p*-nitroaniline (pNa), after its cleavage from the labelled caspase substrates. The values represent the means ± SD of three independent experiments performed in duplicate, n=6 (p<0.0001 TRAIL + artepillin C compared to TRAIL or artepillin C alone as shown by ANOVA).

LNCaP cells were treated with 100 ng/ml TRAIL and/or 50–100 *μ*M artepillin C for the indicated period of time. The stimulation of death receptors induces DISC formation, resulting in the recruitment and activation of caspase-8. TRAIL and artepillin C alone weakly activated caspase-8 in cancer cells. The simultaneous incubation of LNCaP cells with TRAIL and artepillin C significantly increased the caspase-8 activity (Fig. 5A). Caspase-3 is an effector caspase that plays a central role in apoptosis. Co-treatment of LNCaP cells with TRAIL and artepillin C markedly enhanced caspase-3 activity compared to treatment with TRAIL or artepillin C alone (Fig. 5B). The use of the pan-caspase inhibitor, Z-VAD-FMK, the caspase-8 inhibitor, Z-IETD-FMK, or the caspase-3 inhibitor, Z-DEVD-FMK, completely blocked the subsequent cell death induced by TRAIL in combination with artepillin C. These results suggest that artepillin C promotes TRAIL-mediated apoptosis through a caspase cascade.

### Effects of TRAIL and artepillin C on ΔΨm in LNCaP cells

**Figure 6:**
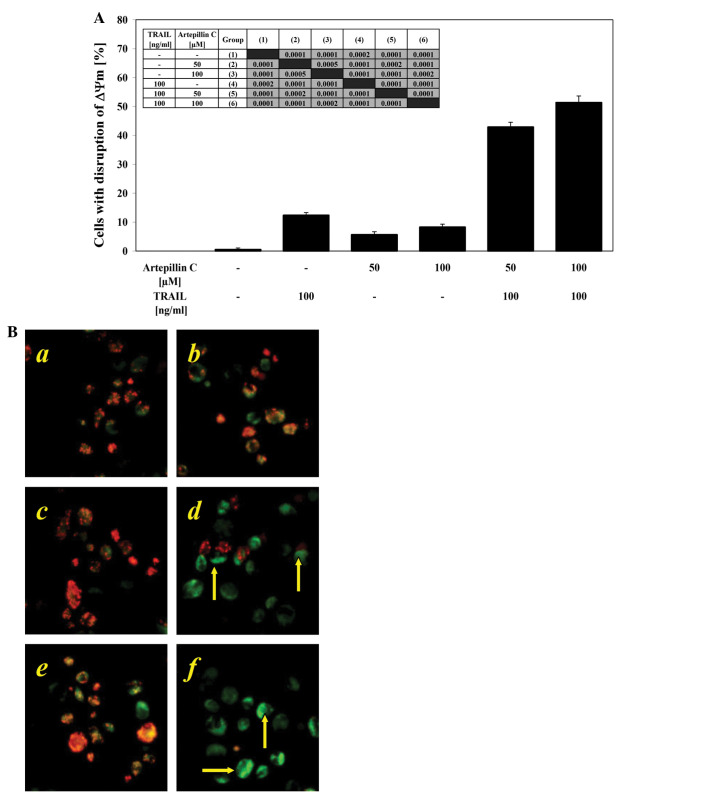
Effects of TRAIL in combination with artepillin C on the mitochondrial membrane potential (ΔΨm) in LNCaP prostate cancer cells. Cells were incubated for 24 h with 100 ng/ml TRAIL and/or 50–100 *μ*M artepillin C. The values represent the meana ± SD of three independent experiments performed in duplicate, n=6 (p<0.001 TRAIL + artepillin C compared to TRAIL or artepillin C alone as shown by ANOVA). (A) TRAIL in combination with artepillin C induced a loss of ΔΨm in LNCaP cells. (B) Disruption of ΔΨm in LNCaP cells was assessed by fluorescent microscopic analysis of DePsipher staining using the following treatment conditions: (a) control cells, (b) cells incubated with 100 ng/ml TRAIL, (c) cells incubated with 50 *μ*M artepillin C, (d) cells incubated with 100 ng/ml TRAIL and 50 *μ*M artepillin C, (e) incubated with 100 *μ*M artepillin C and (f) cells incubated with 100 ng/ml TRAIL and 100 *μ*M artepillin C. Red fluo rescence is emitted from aggregates of DePsipher, which are formed within the mitochondria of healthy cells. Green fluorescence reveals the monomeric form of the DePsipher molecule, which appears in the cytosol after mitochondrial membrane depolarisation (indicated by arrows).

Mitochondrial membrane depolarization is one of the first intracellular changes that occur after the onset of apoptosis. We evaluated whether artepillin C sensitizes cancer cells to TRAIL-induced mitochondrial dysfunction. When the LNCaP cells were treated with 100 ng/ml TRAIL or 50–100 *μ*M artepillin C alone, there was little effect on ΔΨm (12.4±0.9% and 5.6±1.1–8.3±1.0%, respectively). The combination treatment of TRAIL with artepillin C enhanced the loss of ΔΨm in a large percentage of the cells (42.9±1.6–51.4±2.3%) and induced a significant disruption of the ΔΨm (Fig. 6A and B). These results demonstrate that the intrinsic apoptotic pathway is engaged in LNCaP cells treated with both TRAIL and artepillin C.

### Effects of artepillin C and TRAIL on NF-κB activity in LNCaP cells

**Figure 7:**
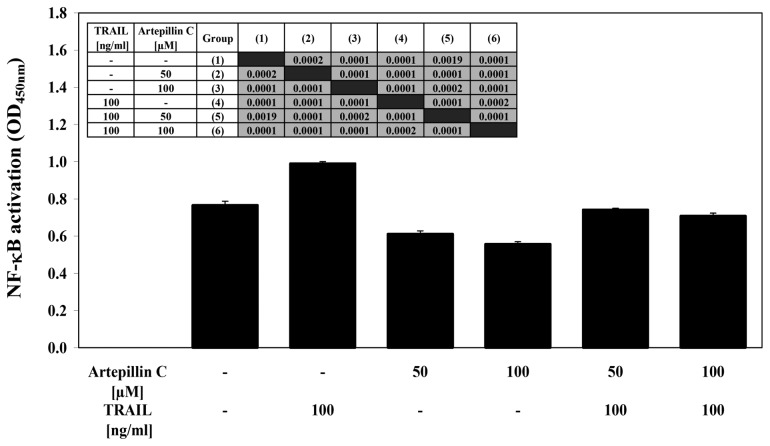
Effects of artepillin C and TRAIL on NF-κB activity in LNCaP prostate cancer cells. Cells were incubated for 24 h with 50–100 *μ*M artepillin C and/or 100 ng/ml TRAIL. The values represent the means ± SD of three independent experiments performed in duplicate, n=6 (p<0.0001 TRAIL + artepillin C compared to TRAIL or artepillin C alone as shown by ANOVA). The effects of artepillin C and/or TRAIL on the NF-κB (p65) binding activity in LNCaP nuclear extracts were measured using the ELISA-based TransAM NF-κB assay.

We examined the effect of artepillin C and/or TRAIL on NF-κB activation in LNCaP cells (Fig. 7). Using the ELISA-based TransAM NF-κB test, we determined the binding activity of the p65 subunit in LNCaP nuclear extracts. Treatment with artepillin C decreased the activity of NF-κB compared with the control cells. By contrast, TRAIL induced the activation of NF-κB in the LNCaP cells. The co-treatment of artepillin C with TRAIL also significantly decreased the NF-κB activity. This shows that artepillin C can help cells overcome TRAIL resistance in LNCaP cells by blocking the NF-κB activation induced by TRAIL.

## Discussion

Propolis contains various phenolic compounds and exhibits a broad spectrum of biological activities (16,36,47). The composition of propolis is complex and largely depends on the geographical origin and specific flora at the site of its collection (17,47). *Baccharis dracunculiforia* is the main botanical source of resins for the green propolis rich in artepillin C (21–23). Phenolic ingredients contribute to the overall cancer preventive and antitumour effects of propolis (16,36). Therefore, propolis is a promising raw mixture of natural compounds that should be studied to discover new pharmaceutical products with chemopreventive properties.

Artepillin C has shown a marked activity against different tumour cells *in vitro*, and it affects cancer cells by inhibiting cell growth and inducing apoptosis (24–26). Akao *et al* demonstrated the suppression of tumour cell growth by artepillin C and two other cinnamic acid derivatives detected in propolis, drupanin and baccharin (51). In our study, artepillin C exerted low direct cytotoxic and apoptotic effects on LNCaP cells. Numerous tests have confirmed that the LNCaP prostate cancer cell line is resistant to TRAIL-mediated apoptosis (15–20). Inactivation of the TRAIL pathway and escape from the TRAIL-mediated immunosurveillance may play important roles in tumour onset and progression (2). Previous studies have shown that TRAIL in combination with propolis extracts or phenolic compounds identified in propolis results in the synergistic induction of cancer cell death (16,17,47). We then treated the TRAIL-resistant LNCaP cells with a combination of TRAIL and artepillin C. The tested compound significantly augmented TRAIL-induced death in the prostate cancer cells. These results suggest that artepillin C exhibits mainly indirect antitumour action by stimulating the TRAIL-mediated apoptotic pathway.

TRAIL triggers a pro-apoptotic signal by binding to the death receptors. The inactivation of TRAIL-R2 (also known as DR5) significantly increases tumour growth *in vitro* and *in vivo*. Thus, the expression of TRAIL-R2 may contribute to the tumour selective induction of apoptosis mediated by TRAIL (1–5). We found that the artepillin C-mediated sensitization of LNCaP cells to TRAIL-induced apoptosis is associated with the upregulation of TRAIL-R2 expression. Indeed, other phenolic compounds isolated from propolis can evoke similar increases in the cell surface expression of TRAIL-R2. Apigenin, kaempferol and quercetin, all reverse TRAIL resistance in cancer cells by influencing TRAIL-R2 levels (12,13,52).

Caspases are crucial players in the induction of apoptosis. TRAIL-mediated apoptosis is primarily executed by the extrinsic death receptor pathway. This pathway involves caspase-8 as the initiator and caspase-3 as the executor (2,19). We observed that co-treatment with TRAIL and artepillin C resulted in the significant activation of caspase-8 and caspase-3 in LNCaP cells, whereas treatment with TRAIL or artepillin C alone weakly activated both caspases. Previous studies have reported that TRAIL induces caspase cleavage when combined with propolis constituents (apigenin, chrysin, kaempferol and quercetin) in cancer cells (12,13,52,53). The present study confirms that the sensitization of prostate cancer cells to TRAIL by artepillin C is accomplished through an extrinsic, receptor-and caspase-dependent pathway.

Mitochondrial dysfunction is considered a hallmark of apoptosis (14,18,19). Further analysis of the ΔΨm showed that the TRAIL and artepillin C co-treatment affected the intrinsic pathway in LNCaP cells via a significant reduction in ΔΨm compared to treatment with TRAIL or artepillin C alone.

In addition to defects in the extrinsic and intrinsic apoptotic pathways, the NF-κB survival pathway in tumour cells may be responsible for the failure to undergo apoptosis (19,20,54). The activation of NF-κB in LNCaP cells leads to TRAIL resistance, while the downregulation of NF-κB by artepillin C sensitizes cancer cells to TRAIL-mediated death.

To our knowledge, in this study, we show for the first time the mechanisms by which artepillin C helps cancer cells to overcome TRAIL resistance. Artepillin C achieves this through the upregulation of the TRAIL-R2 receptor, activation of caspase-8 and caspase-3, the loss of ΔΨm and the downregulation of NF-κB. Artepillin C as the main phenolic compound isolated from Brazilian green propolis sensitizes prostate cancer cells to TRAIL-induced apoptosis, engaging similar cellular targets in LNCaP cells, such as those induced by EEP (17). The evidence confirms that of all the phenolic compounds in Brazilian green propolis, artepillin C is predominantly responsible for the action of propolis on TRAIL-mediated apoptosis in cancer cells.

These results define the cancer chemopreventive action of artepillin C through the modulation of TRAIL-mediated apoptotic signalling pathways. We hypothesize that artepillin C supports TRAIL-mediated immune defense against cancer cells and may therefore represent a prostate cancer immunochemopreventive agent.
